# Graphene Enhances Actin Filament Assembly Kinetics and Modulates NIH-3T3 Fibroblast Cell Spreading

**DOI:** 10.3390/ijms23010509

**Published:** 2022-01-03

**Authors:** Jinho Park, Pavlo Kravchuk, Adithi Krishnaprasad, Tania Roy, Ellen Hyeran Kang

**Affiliations:** 1 NanoScience Technology Center, University of Central Florida, Orlando, FL 32826, USA; Jinho.Park@knights.ucf.edu (J.P.); Pavlo@knights.ucf.edu (P.K.); adithiprasad@Knights.ucf.edu (A.K.); tania.roy@ucf.edu (T.R.); 2 Department of Materials Science and Engineering, University of Central Florida, Orlando, FL 32816, USA; 3 Department of Electrical and Computer Engineering, University of Central Florida, Orlando, FL 32816, USA; 4 Department of Physics, University of Central Florida, Orlando, FL 32816, USA

**Keywords:** actin cytoskeleton, assembly kinetics, graphene, excluded area, cell spreading

## Abstract

Actin plays critical roles in various cellular functions, including cell morphogenesis, differentiation, and movement. The assembly of actin monomers into double-helical filaments is regulated in surrounding microenvironments. Graphene is an attractive nanomaterial that has been used in various biomaterial applications, such as drug delivery cargo and scaffold for cells, due to its unique physical and chemical properties. Although several studies have shown the potential effects of graphene on actin at the cellular level, the direct influence of graphene on actin filament dynamics has not been studied. Here, we investigate the effects of graphene on actin assembly kinetics using spectroscopy and total internal reflection fluorescence microscopy. We demonstrate that graphene enhances the rates of actin filament growth in a concentration-dependent manner. Furthermore, cell morphology and spreading are modulated in mouse embryo fibroblast NIH-3T3 cultured on a graphene surface without significantly affecting cell viability. Taken together, these results suggest that graphene may have a direct impact on actin cytoskeleton remodeling.

## 1. Introduction

Actin is an essential cytoskeletal protein that promotes the reorganization of cellular architectures, thereby enabling cell morphogenesis, migration, and differentiation [1,2,3]. Actin monomers polymerize into double-stranded helical filaments in the presence of cations and ATP hydrolysis. [4,5,6]. Within cells, actin filament assembly dynamics are tightly regulated by various intracellular environmental factors, including cation interactions [7,8], macromolecular crowding [9,10,11], and actin binding proteins [6,12,13]. Changes in the microenvironment have also been also shown to affect the actin cytoskeleton. Recent studies have suggested that exposure to nanomaterials, in particular carbon-based nanomaterials (i.e., single-wall carbon nanotubes (SWCNT), graphene oxide, and graphene), can modulate actin polymerization [14,15,16,17,18].

Graphene is a single layer of sp^2^ hybridized carbon nanomaterial that has served as a promising biomaterial due to its unique structural, mechanical, thermal, and electrical properties [19,20,21,22] as well as biocompatibility [23,24,25]. These properties render graphene an effective nanomaterial that can be used in drug delivery vehicles [26], biosensing [27], cancer therapy [28], and scaffolds for tissue engineering [29]. Increasing usage of graphene for cellular applications requires understanding how the physicochemical properties of graphene affect biomolecular interaction, adsorption, and conformation [25], thus potentially affecting cellular processes. Given that graphene potentially interacts with the actin cytoskeleton, it is important to understand how graphene affects actin filament assembly dynamics for proper biomedical applications.

Several studies have shown that graphene can alter the actin cytoskeleton at the cellular level indirectly through triggering the signaling pathway [30], producing reactive oxygen species (ROS) [31,32], and reducing ATP production [33]. Graphene has been shown to impact actin rearrangement in naive macrophages by enhancing cytokine and chemokine production, decreasing cell adhesion [30]. Accumulation of graphene nanoflakes on the cell membrane of Vero cells (monkey kidney cells) causes ROS production, followed by actin filament rearrangement [31]. Zhou et al. reported that upon cellular uptake, graphene nanoflakes disrupted the electron transfer in mitochondria and reduced ATP production, which may lead to impaired actin filament assembly in breast cancer cells [33]. In addition, a molecular dynamics (MD) simulation has shown that actin monomers adhere to graphene via weak interactions, including van der Waal forces, electrostatic interactions, and hydrogen bonding [34]. These interactions are not strong enough to dissociate two actin monomers compared to graphene oxide (GO) and reduced graphene oxide (rGO) [16,17], of which functional groups on graphene oxide can form hydrogen bonds with oxygen-containing residues in actin [34], thereby inducing actin disassembly [16,17]. While the effects of graphene on the actin cytoskeleton have been reported at the cellular level, how graphene modulates actin filament assembly is not well established.

In this study, we investigate how graphene modulates actin filament assembly kinetics utilizing total internal reflection fluorescence (TIRF) microscopy imaging and bulk pyrene fluorescence assay. We hypothesize that the non-covalent interaction between actin and graphene may have a direct impact on actin filament assembly. We demonstrate that both pure graphene flakes and graphene surface enhance the average growth rates of individual actin filaments in a concentration-dependent manner. Furthermore, we demonstrate mouse embryo fibroblast NIH-3T3 cells seeded on the graphene surface exhibit stretched cell peripheral regions without incurring cytotoxicity, indicating the hydrophobicity of the graphene surface may modulate cellular morphology. Taken together, our study suggests that graphene can directly modulate actin assembly kinetics, thereby potentially modulating the actin cytoskeleton remodeling in cells.

## 2. Results and Discussions

### 2.1. Graphene Flakes Affect Actin Filament Length without Hampering Polymerization

**Figure 1:**
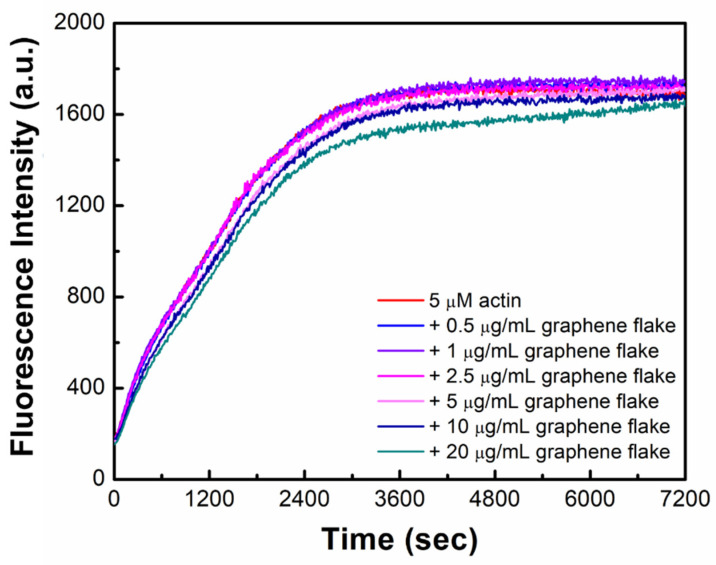
Graphene flakes do not hamper bulk actin polymerization. Actin (5 µM, 20% pyrene-labeled) polymerization in the presence of graphene flakes at varying concentrations (0.5–20 μg/mL) was monitored. Data are a representative from triplicated trials.

We first evaluated the effects of graphene flakes on steady-state actin polymerization using TIRF microscopy imaging. We chose pristine graphene flakes rather than graphene oxide flakes or its derivatives since pure graphene flakes have well-defined hydrophilic and hydrophobic regions, demonstrated by a recent study [35]. We polymerized actin filaments (18–20% Alexa-labeled) in the absence (control) or presence of graphene flakes (0.5–20 μg/mL), and then measured the steady-state actin filament lengths in the presence of graphene flakes (0.5–20 μg/mL) (Figure S1). The average filament lengths (control, 3.26 μm) significantly increased (~23.9%) with 5 μg/mL graphene flakes (*L_a_*_vg_ = 4.04 μm) where the filament length peaked among the range of graphene flake concentrations. At 10 μg/mL graphene flakes, similar lengths of actin filaments compared to controls were observed (*L_a_*_vg_ = 3.02 μm), and 20 μg/mL graphene flakes reduced the filament lengths by 15% compared to controls. Next, we conducted bulk kinetic assays using pyrene-labeled actin to determine the effects of graphene flakes on actin polymerization (Figure 1). In the presence of graphene flakes, the pyrene fluorescence intensity increased in comparison to controls, indicating graphene flakes do not hamper the assembly of actin monomers into filamentous actin (Figure 1). The highest concentration of graphene flakes (20 μg/mL) was slightly delayed in reaching a similar maximum fluorescence intensity compared to controls. (Figure 1). These data indicate that graphene flakes result in modulation of filament lengths without hampering bulk actin polymerization.

### 2.2. Graphene Flakes Modulate the Rates of Actin Filament Elongation

**Figure 2:**
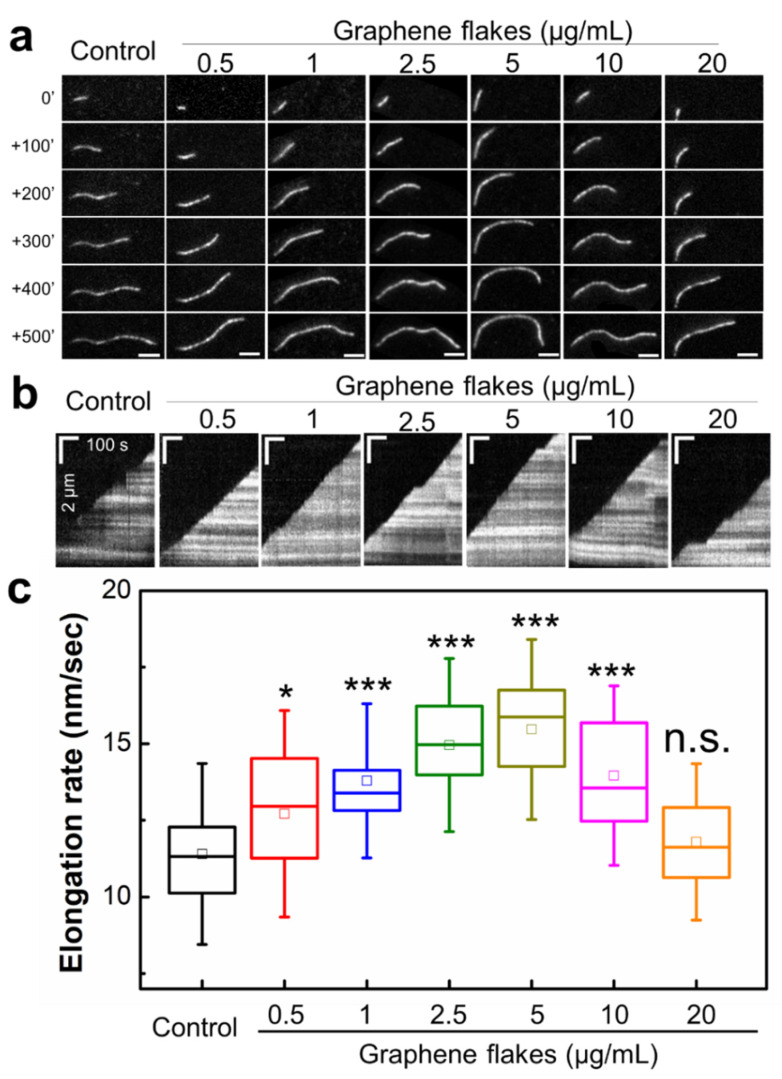
Graphene flakes modulate the assembly kinetics of individual actin filaments. (**a**) Representative total internal reflection fluorescence (TIRF) microscopy images of growing actin filaments in the presence of varying concentrations of graphene flakes (0.5–20 μg/ml) (Δt = 100 s). (**b**) Linear kymographs of the growing filaments in the presence of graphene flakes. Alexa-labeled actin monomers were polymerized in a functionalized flow cell. Images of actin filaments were taken every 5 s. Length scale bar (vertical) is 2 μm, and time scale bar (horizontal) is 100 s. (**c**) The rates of actin filament elongation in the presence of graphene flakes at varying concentrations. The elongation rate was determined by the slope of actin filament length over time, and then converted to a function of time to nm. The box represents the 25–75th percentile, whiskers indicate standard deviation (SD), and the middle square is the mean. Statistical analysis was performed using Tukey’s test. *N* = 24–67, n.s; not significant, *; *p* < 0.05, ***; *p* < 0.001.

To determine how pure graphene flakes in solution affect actin filament assembly rates, we visualized the growth of individual actin filaments with varying concentrations of graphene flakes using a functionalized flow cell chamber (see Materials and Method for details). We polymerized actin filaments (18–20% Alexa-labeled, 0.38% biotinylated) in the absence (control) or presence of graphene flakes (0.5–20 μg/mL). The presence of graphene flakes rendered faster actin filament elongation (Figure 2a,b, Videos S1–S7). Without graphene flakes, the elongation rate of actin was 11.40 ± 1.97 nm/s (4.22 ± 0.77 subunits/s), which is a similar value with the previously reported value (3.9 ± 0.4 subunits/s [36]) (Figure 2c). A total of 0.5–5 μg/mL graphene flakes resulted in statistically significant increases in the average elongation rates up to 15.46 ± 1.96 nm/s, which is approximately an 11–36% increase compared to controls. With 10 μg/mL graphene flakes, the extent of increase in elongation rate dropped to 13.96 ± 1.96 nm/s but was still significantly higher than controls (22% increase). With 20 μg/mL graphene flakes, the elongation rate did not show a significant difference (11.79 ± 1.70 nm/s).

Actin assembly dynamics are controlled by various physiological factors [6,37,38]. One such physiological factor affecting actin polymerization is macromolecular crowding that induces the excluded volume effect [10]. In a crowded environment, the volume accessible for actin monomers is excluded from the space occupied by macromolecules. The excluded volume effect increases the rates of reactions, including protein assembly and protein–protein interactions [39,40]. The presence of crowding reduces the critical concentration of ADP-actin and enhances filament stability by reducing subunit dissociation [10,41]. The excluded volume effect is applied in three-dimensional materials, however, it can also exist in terms of two-dimensional materials because two-dimensional materials have an exclusion region around each particle, unless all particles overlap [42]. As graphene flakes exclude the region available for actin monomers, the critical concentration for actin may be shifted modulating the actin assembly kinetics. 

The observed reduction in actin polymerization at higher concentration of graphene flakes (20 µg/mL) can be explained by the location of two adjacent graphene flakes. The excluded area of two-dimensional materials depends on the relative angle between two particles [42]. The excluded area is minimized when two particles are in parallel or antiparallel (the relative angle between two particles is 0 or π) [42]. High concentrations of graphene flakes may increase the possibility of encountering two particles face-to-face, thereby decreasing the excluded area effect. Unlike three-dimensional spherical nanoparticles, which only make a point contact that allow them to retain most of their individual surface areas [43], two-dimensional nanomaterials may exhibit more drastic effects.

### 2.3. Graphene Surface Accelerates Actin Filament Elongation

**Figure 3:**
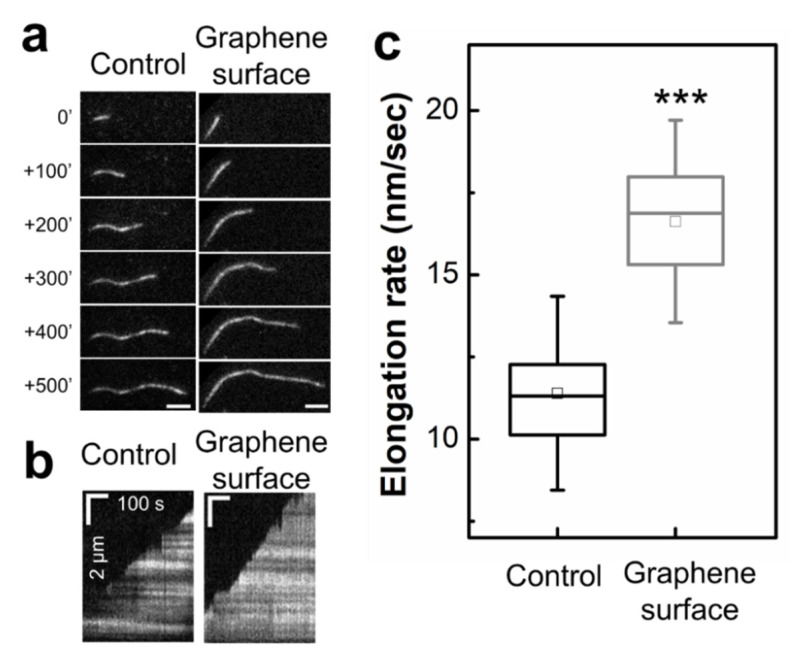
Graphene surface increases the rates of individual actin filament elongation. (**a**) Representative TIRF microscopy images of growing actin filaments (control or on graphene surface), Δt = 100 s. (**b**) Linear kymographs of the growing filaments on a graphene surface. Alexa-labeled actin monomers were polymerized in a functionalized flow cell. Images of actin filaments were taken every 5 s. Length scale bar (vertical) is 2 μm, and time scale bar (horizontal) is 100 s. (**c**) The rates of actin filament elongation were measured for control samples or for filaments assembled on the graphene surface that was functionalized by pyrenebutyric acid *N*-hydroxysuccinimide ester (PNHS). The elongation rates were determined by the slope of the actin filament lengths over time, and then converted to a function of time to nm. The box represents the 25–75th percentile, whiskers indicate standard deviation (SD), and the middle square is the mean. Statistical analysis was performed using Tukey’s test. *N* = 24–67, ***; *p* < 0.001.

We further investigated the assembly of actin on a graphene surface. We used a graphene layer-transferred flow cell to allow consistent interactions between actin monomers and graphene (see Materials and Methods). Interestingly, the graphene surface significantly enhanced the rates of actin filament elongation (16.62 ± 2.05 nm/s), approximately a 45% increase compared to those of the control samples (Figure 3).

Self-assembly of peptides and proteins into specific organized structures is joined by non-covalent bonds [44]. Self-assembly of biomolecules on the surface of 2D materials can be influenced by the interaction at the interface, which depends on the surface properties, such as hydrophilicity/hydrophobicity, electrostatic interactions, and physical properties including topography and roughness [45]. For example, hydrophobic and rough graphene surfaces accelerated the nucleation of insulin amyloid fibrils compared to hydrophilic and smooth surfaces since fibrils tend to assemble into the adsorbed protein on the surface, resulting in faster fibril formation [46]. In addition, the interaction at the interface should properly maintain the structure and functions of the assembled organizations without any disruptions. Hydrophilic surfaces may allow biomolecules to adsorb more strongly on the surfaces via hydrophilic interactions and hydrogen bonding, which may hamper the assembly of biomolecules. Indeed, two residues in actin (GLU270 and LYS284) can form hydrogen bonds with epoxy groups on GO, resulting in a more stable structure compared to pristine graphene [34]. The interaction between GO and two actin dimers is strong enough to dissociate them and induce changes in the secondary structure of actin, thereby disrupting the formation of actin filaments [16]. Thus, interactions with a graphene surface may accelerate actin assembly due to its hydrophobicity.

### 2.4. Non-Cytotoxic Graphene Surface Alters Morphology of Mouse Embryo Fibroblasts

**Figure 4:**
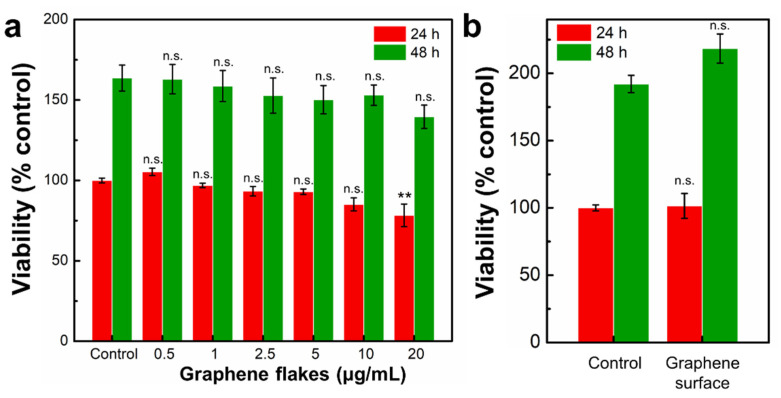
Effects of graphene flakes on viability of NIH-3T3 cells. The viability of NIH-3T3 cells was determined after 24 h and 48 h exposure to various concentrations of (**a**) graphene flakes (0.5–20 μg/mL) or (**b**) on a graphene surface using a WST-1 assay. The results are expressed as the mean ± standard deviation (S.D.) of three independent experiments. n.s., not significant; **, *p* < 0.01.

We further evaluated the effects of graphene on cytotoxicity of mouse embryo fibroblast NIH-3T3 cells, which are widely used to determine the effects of materials on cellular behaviors [47,48]. NIH-3T3 cells were treated with graphene flakes or seeded on a graphene surface, and their viability was determined using a WST-1 assay. Our WST-1 assay results showed graphene flakes in the range of 0.5–10 μg/mL were not cytotoxic compared to controls (Figure 4a). A total of 20 μg/mL graphene flakes showed significant difference in cell viability (from controls) after 24 h (Figure 4a); however, the cell viability at 48 h did not show any significant difference, indicating that the highest concentration of graphene flakes (20 μg/mL) is not cytotoxic to NIH-3T3 cells (Figure 4a). Several studies have reported that graphene flakes in suspension could be cytotoxic because they can accumulate and cover the cell surface or form pores on the cell membrane [49,50]. However, our results indicate that graphene flakes have minimal impact on cell viability. The graphene surface also did not show any significant difference with controls (Figure 4b), which is in good agreement with observations for other cells, such as mouse embryo BALB/3T3 cells and murine fibroblast L929 cells [51,52]. Of note, the cytotoxicity could be varying depending on the cell type. For example, 25 μg/mL of graphene flakes induced cytotoxicity to human skin fibroblasts [50]. A549 cells exhibited cytotoxicity starting from 50 μg/mL of graphene oxide sheets, whereas over 10 μg/mL of graphene oxide sheets were cytotoxic to Raw549 cells [49].

**Figure 5:**
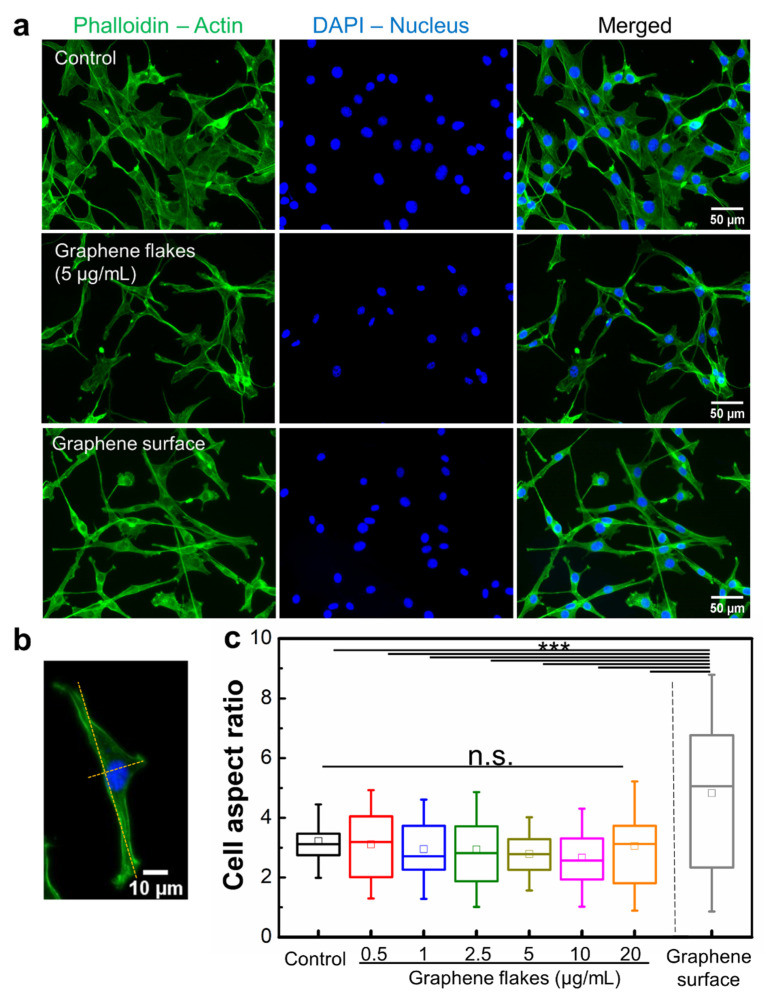
Effects of graphene flakes and graphene surface on NIH-3T3 cell morphology and spreading. (**a**) NIH-3T3 cells were incubated for 24 h on either a poly-L-lysine coated coverslip (top), on a poly-L-lysine coated coverslip and treated with graphene flakes (5 μg/mL) (middle), or on a pristine graphene monolayer (bottom). Then, cells were stained with Acti-stain 488 phalloidin (actin, green) and DAPI (nucleus, blue). (**b**) Representative confocal microscopy image of a cell used to measure the cell aspect ratio (major/minor axis). (**c**) Quantified cell aspect ratio of NIH-3T3 cells incubated with graphene flakes or on a graphene surface. *N* = 20–73 across three independent experiments. n.s., not significant; ***, *p* < 0.001.

Next, we investigated how graphene affects cell morphology and spreading by visualizing the actin cytoskeleton inside NIH-3T3 cells with optical and confocal microscopy (Figure 5a, Figures S2 and S3). Optical and fluorescence imaging revealed graphene flakes did not induce any morphological changes in NIH-3T3 cells compared with controls (Figure 5a, Figures S2 and S3). In contrast, cells cultured on the graphene surface were observed to be stretched (Figure 5a). We further quantified the stretched cell morphology by analyzing the cell aspect ratio (i.e., ratio between long axis and short axis) as described previously [53] (Figure 5b). The average cell aspect ratio of NIH-3T3 cells without graphene was 3.22 ± 0.82, which is within the range of the previously reported values (1.3–3.8) [53,54]. The average cell aspect ratio in the presence of graphene flakes (0.5–20 μg/mL) did not show significant differences compared to control samples (Figure 5c, Figure S2). Interestingly, cells seeded on the graphene surface exhibited the widest range of cell aspect ratios (4.83 ± 2.64) (Figure 5c). Both highly elongated and unspread cell morphology was reflected in the large standard deviation of the cell aspect ratio. This morphological analysis suggests that interactions with graphene surfaces can modulate cell shapes as well as cell spreading.

Cellular adhesion and spreading can be altered by surface properties, such as topography and wettability (hydrophobicity and hydrophilicity) [55,56]. Lee et al. have shown that single graphene layer-transferred glass surfaces exhibit higher nano-roughness compared to glass alone, thereby enhancing the adhesion of human embryo stem cells with increased focal adhesion [57]. Another surface physical factor affecting cell adhesion is hydrophobicity and hydrophilicity of the surface [58]. The water contact angle on a plain glass surface is 42°, while on a graphene layer-transferred glass surface is 67° [59]. Optimal hydrophobicity is varied and strongly depends on the type of cells. For example, mouse fibroblast L cells showed maximum cell adhesion on polymer-coated surfaces where the contact angle ranges between 60° to 80° [60]. The optimal adhesion of osteosarcoma cells occurs at the water contact angle of siloxane-coated polystyrene, 64° [61]. These factors may modulate the adhesion of NIH-3T3 cells on graphene layer-transferred glass with morphological changes, evidenced by changes in spreading (Figure 5). The changes in cell spreading and adhesion induced by external physical cues are accompanied with the actin cytoskeleton modulation. Cell spreading is an active process that is controlled by complex mechanisms, including actin polymerization, cell membrane deformation, and interaction at the interface of a cell membrane-substrate [62]. Therefore, it is possible that the regulation of the intracellular cytoskeleton is a result of adaptation to the microenvironment as cells sense and respond to the mechanical features of a graphene surface. 

## 3. Materials and Methods

### 3.1. Sample Preparation

Actin was purified from rabbit skeletal muscle acetone powder (Pel-Freeze Biologicals Inc., Rogers, AR, USA) and gel-filtered over Sephacryl S-300 equilibrated in buffer A (2 mM Tris-HCl, 0.2 mM CaCl_2_, 1 mM NaN_3_, 0.2 mM ATP, 0.5 mM DTT, pH 8.0) as described in [63]. Purified actin was labeled with Alexa-488 succimidyl ester dye (Molecular Probes Inc., Eugene, OR, USA) (the labeling efficiency was ~ 40%) as described in [7]. Ca^2+^-actin monomers were converted into Mg^2+^-actin monomers by the addition of 0.2 mM EGTA and MgCl_2_ with the concentration of actin plus 10 μM for 5 min, then 1/10th volume of 10× polymerization buffer (500 mM KCl, 20 mM MgCl_2_, 100 mM imidazole, pH 7.0, 10 mM ATP, and 10 mM DTT) was added and incubated at room temperature (T ~ 22 °C) for 1 h as described in [64].

Graphene flakes were purchased from Alfa Aesar Co., Inc. (Tewksbury, MA, USA) (surface area 500 m^2^/g, 5 nm thickness, diameter ~ 2 μm). Graphene flakes were dissolved in ddH_2_O and vortexed for 30 s before use for in vitro actin polymerization or dissolved in DMEM media for the cell experiment. A pristine graphene sheet (10 mm × 20 mm) was transferred on a 22 mm × 40 mm coverslip. Chemical vapor deposition (CVD) graphene grown on copper foil from Grolltex (San Diego, CA, USA) was used. CVD grown monolayer graphene was wet transferred [65] on the cover slip. After the transfer, the samples were annealed in forming gas (90 % N_2_ and 10 % H_2_) ambient at 400 °C for 3 h to remove the contaminants on the graphene film.

### 3.2. Steady-State TIRF Microscopy Imaging and Data Analysis

For steady-state imaging, actin monomers (12–18% Alexa-labeled, 1 μM) were polymerized by the addition of KMI buffer in the presence of graphene flakes (dissolved in ddH_2_O, dilution factor × 50) for 1 h at room temperature. F-actin samples were diluted in optical imaging buffer (10 mM imidazole pH 7.0, 50 mM KCl, 2 mM MgCl_2_, 1 mM ATP, 1 mM DTT, 15 mM glucose, 1 mg/mL catalase, and 0.2 mg/mL glucose oxidase) [64,66]. Actin filaments were immobilized on coverslips coated with 0.01% *v*/*v* poly-L-lysine (Sigma-Aldrich, St. Louis, MO, USA), which produces a weak electrostatic force to adhere biopolymers to glass surfaces [66]. F-actin images were acquired at room temperature using a Nikon Eclipse Ti TIRF microscope equipped with a Hamamatsu Image EM X2 CCD camera, 100× oil immersion objective (numerical aperture 1.49), and Nikon LU-N4 laser. Nikon Imaging Software (NIS) Elements (ver. 5.02) was used to capture images (pixel size = 0.16 μm/pixel). F-actin lengths (*L*) were analyzed using ImageJ, Persistence [67], and OriginLab (ver. 8.5).

### 3.3. Pyrene Assay

Pyrene actin (>99% purity) was purchased from Cytoskeleton Inc. (Denver, CO, USA) and mixed with unlabeled actin monomers to make 20% labeled pyrene actin. Ca^2+^-bound pyrene labeled G-actin was exchanged to Mg^2+^ as previously described in [68]. Graphene flakes (dissolved in ddH_2_O, dilution factor ×100) were then added to Mg^2+^-G-actin. In total, 1/10th volume of 10× KMI buffer was rapidly added to pyrene actin (5 µM) to start actin polymerization. Pyrene fluorescence was monitored at 407 nm every 10 s over 2 h in a 96-well fluorescence plate reader (SpectraMax Gemini XPS, Molecular Devices LLC, CA, USA) with 360 nm excitation. Since the graphene surface layer cannot be directly transferred into a 96-well plate, it was not possible to observe pyrene–actin polymerization on the graphene-transferred surface.

### 3.4. Flow Cell Preparation and Real-Time TIRF Microscopy Imaging

Functionalized coverslips were prepared using a modified protocol based on Winterhoff et al. [68,69]. Briefly, coverslips were sonicated at 60 °C for 45 min in 1 M KOH, 1 M HCl, and 70% ethanol. After each wash, the slides were rinsed with 60 °C ddH_2_O. Two functionalization solutions were prepared by using an 80% ethanol and 1% 1 M HCl solution. In total, 1 mg/mL solutions of mPEG-silane (MW 2000 Da, Laysan Bio Inc., Arab, AL, USA) and biotin-PEG-silane (MW 3400 Da, Laysan Bio Inc., Arab, AL, USA) were prepared in 80% ethanol. The solutions were mixed at a ratio of 1:500 of biotin-PEG-silane to mPEG-silane. Coverslips were incubated overnight at 60 °C in a humid chamber, rinsed with warm water, and dried before using. Then, a flow cell chamber was constructed as described in [69]. A 1% (*w*/*v*) fatty-free BSA solution containing 1% streptavidin (in 1X KMI buffer) was injected into the flow cell chamber to block unnecessary binding. Images of polymerizing actin (18–20% Alexa-labeled) were taken every five seconds using a Nikon Eclipse Ti TIRF microscope equipped with a Hamamatsu Orca-Flash 4.0 Digital Camera C13440, 100× oil immersion objective (numerical aperture 1.49), and Nikon LU-N4 laser. Nikon Imaging Software (NIS) Elements (ver. 5.02) was used to capture images (pixel size = 0.07 μm/pixel).

From the stacks of images of polymerizing actin filaments, we obtained kymographs that showed the changes in fluorescence intensity of the actin filament backbone over the course of time using ImageJ software. We calculated the actin filament growth rates by plotting the time frame versus the elongated filament length, where the slope yields the elongation rate of individual actin filaments [36,69].

A graphene layer-transferred coverslip was functionalized by pyrenebutyric acid *N*-hydroxysuccinimide ester (PNHS) dissolved in dimethylformaide (DMF) for 24 h since biotin-PEG was not able to anchor onto the graphene surface due to its hydrophobicity. PNHS can be used as a linker of biomolecules and graphene as the pyrene groups interact with the graphene surface via π-π stacking while the succinimidyl ester groups react with amines of biomolecules [70,71]. The hydrodynamic radii of PEG mw 3400 for the silane-PEG-biotin is 1.94 nm [72]. Functionalized graphene surface was washed with DMF and ddH_2_O thoroughly, followed by incubation with the biotin-PEG-silane/mPEG-silane solution (the ratio of biotin-PEG-siline to mPEG-silane 1:500) overnight at 60 °C in a humid chamber, rinsed with warm water, and dried before using.

### 3.5. Cell Culture

Mouse embryo fibroblast NIH-3T3 cells were purchased from American Type Culture Collection (ATCC; Rockville, MD, USA). NIH-3T3 cells were maintained in Dulbecco’s modified Eagle’s medium (DMEM) supplemented with 10% (*v*/*v*) Fetal Calf Serum (FCS) and 1% penicillin/streptomycin in a 5% CO_2_ incubator at 37 °C. NIH-3T3 cells were seeded in a 6-well culture plate or 96-well culture plate for 24 h. Then, graphene flakes in DMEM containing 10% FCS and 1% P/S were treated into cells and incubated up to 48 h.

### 3.6. Cell Viability Assay, Cell Imaging, and Morphology Analysis

Cells were seeded in 96-well plates (Corning Inc., Corning, NY, USA) at a density of 5 × 10^3^ cells/well, with a total volume of 200 μL. After 24 h, cells were treated with graphene flakes and kept at 37 °C in a humidified 5% CO_2_ incubator. After 24 and 48 h, 20 μL of WST-1 reagents (10:1) (Sigma-Aldrich, St. Louis, MO, USA) were supplemented, and cells were incubated for 2 more hours. For the cell viability assay on the graphene surface, cells were seeded on graphene layer-transferred coverslips or poly-L-lysine-coated coverslips (0.01% *v*/*v* poly-L-lysine) as a control in 6-well plates at a density of 1 × 10^5^ cells/well, with a total volume of 2000 μL. After 24 h and 48 h, 200 μL of WST-1 reagents (10:1) (Sigma-Aldrich, St. Louis, MO, USA) were supplemented, and cells were incubated for 2 more hours. Final absorbance of the samples against a background control (medium alone, which also served as a blank) was measured at 450 nm and 630 nm using a SpectraMax i3x (Molecular Devices, Sunnyvale, CA, USA).

Cells were seeded at a density of 1 × 10^5^ cells/well onto coverslips coated with poly-L-lysine (0.01% *v*/*v*) in a 6-well cell culture plate. After 24 h, cells were treated with graphene flakes (0.5–20 μg/mL) for 24–48 h. Cells were also seeded at the same density onto graphene-layer transferred coverslips and incubated for 24 h. Bright field microscopy images of cells were obtained using TS2 inverted microscopy (Nikon, Tokyo, Japan) at 24 h and 48 h after treatment. Prior to obtaining fluorescence microscopy images, cells were fixed with 4% paraformaldehyde for 10 min. The fixed cells were then permeabilized for 5 min with 0.1% Triton X-100 in PBS. Coverslips were rinsed with PBS and incubated for 30 min with Acti-stain 488 Phalloidin (Cytoskeleton Inc., Denver, CO, USA). Following a PBS wash, the coverslips were incubated for 5 min with DAPI (Cytoskeleton Inc., Denver, CO, USA) for counterstaining. Fluorescent images were collected with a BZ-X800 confocal microscope (Keyence, Itasca, IL, USA). To quantify the morphology of stretched cells, the aspect ratios (major axis/minor axis) of cells were measured using the ImageJ program [53].

### 3.7. Statistical Analysis

Statistical significance of the numbers of filaments per bundle, bundle persistence lengths, and bundle diameters was determined using OriginLab ver. 8.5 software by conducting multiple analysis of variance (ANOVA) with post-hoc Tukey’s test. Notation for probability (*p*-value): n.s., not significant (*p* > 0.05); *, *p* < 0.05; **, *p* < 0.01; ***, *p* < 0.001.

## 4. Conclusions

Here, we report the effects of graphene flakes and a graphene surface on actin filament assembly kinetics and NIH-3T3 fibroblast cell spreading as well as morphology. Direct visualization of individual actin filament assembly in real-time demonstrates that both graphene flakes and a graphene surface significantly enhance the rates of actin filament elongation. Cell culture experiment results show that graphene flake uptake does not incur cytotoxicity. Further, interactions with a graphene surface lead to changes in NIH-3T3 cell spreading and stretched cell morphology, which may be related to the enhanced actin filament assembly kinetics. Our work suggests that interaction/interface with graphene may have a direct impact on the actin cytoskeleton remodeling at the molecular level, possibly modulating cell motility and physiology.

## Data Availability

Not applicable.

## Supplementary Materials

The following are available online at https://www.mdpi.com/article/10.3390/ijms23010509/s1.
