# Supplementary material for: Graphene Enhances Actin Filament Assembly Kinetics and Modulates NIH-3T3 Fibroblast Cell Spreading
Source: Int J Mol Sci. 2022 Jan 3;23(1):509. doi: 10.3390/ijms23010509 (PMC8745492; doi:10.3390/ijms23010509)
Supplement: Supplementary file 1 [file ijms-23-00509-s001.zip › ijms-1492338-Supplementary-Final.pdf]

Supplementary Information

Graphene enhances actin filament assembly kinetics and modulates NIH-3T3 fibroblast cell spreading

Jinho Park[1,2], Pavlo Kravchuk[1], Adithi Krishnaprasad[1,3], Tania Roy[1,2,3], and Ellen Hyeran Kang[1,2,4*]

[1]NanoScience Technology Center, [2]Department of Materials Science and Engineering, [3]Department of Electrical and Computer Engineering, [4]Department of Physics, University of Central Florida, Orlando, FL 32816, USA

*Corresponding author: Ellen H. Kang, University of Central Florida, NanoScience Technology Center, 12424 Research Parkway, Orlando, FL 32826, USA. Tel: +1-407-823-2368; E-mail: Ellen.Kang@ucf.edu;

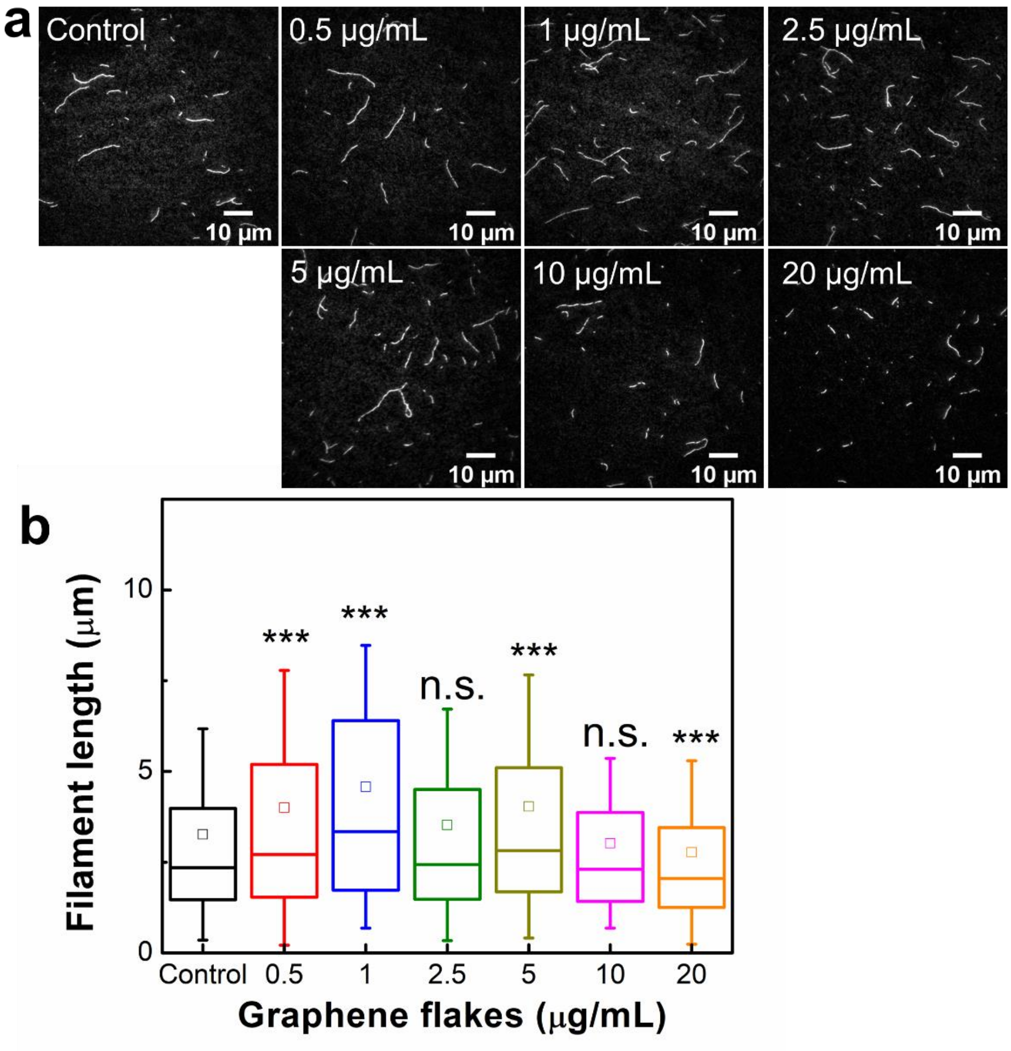

Fig. S1 Graphene flakes modulate steady-state actin filament lengths. (a) Representative TIRF microscopy images of actin filaments (1 µM, Alexa-labeled) in the absence (control) or presence of varying concentrations of graphene flakes (0.5-20 µg/mL) in polymerization buffer (50 mM KCl, 2 mM $MgCl_2$, 10 mM Imidazole, pH 7.0, 1 mM ATP, 1 mM DTT). (Scale bars, 10 µm). (b) Average steady-state actin filament lengths with varying concentrations of graphene flakes. The box represents the 25-75th percentile, whiskers indicate standard deviation (SD), and the middle square is the mean. Statistical analysis was performed using Tukey's test. *N*=979-1669, n.s; not significant, ***; p<0.001.

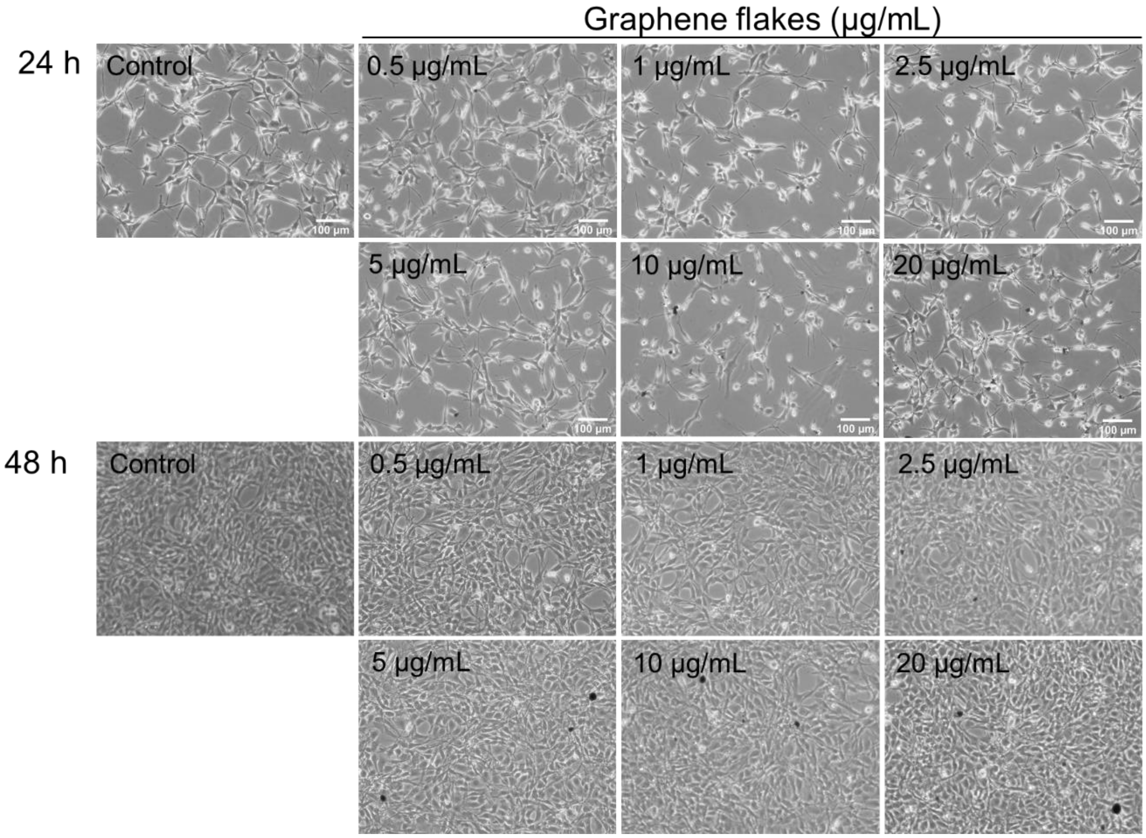

Fig. S2 Optical microscopy images of NIH-3T3 cells with graphene flakes. The bright field images of cells in the absence (control) or presence of varying concentrations (0.5-20 µg/mL) of graphene flakes were taken after 24 h and 48 h of the treatment (Scale bars, 100 µm). Cells were incubated up to 48 h on a poly-L-lysine coated coverslip and treated with graphene flakes, followed by staining with Alexa-488 phalloidin (actin, green) and DAPI (nucleus, blue).

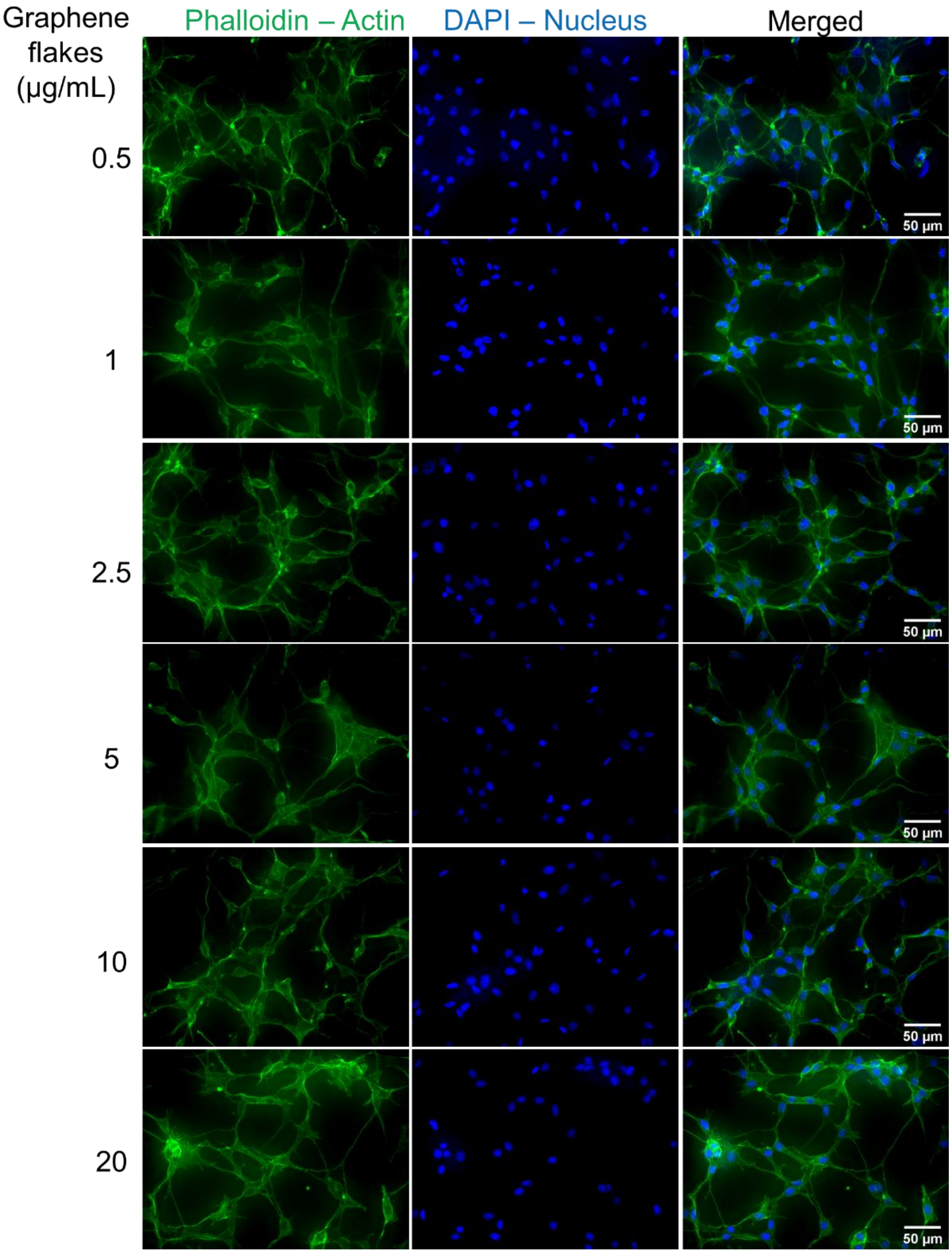

Fig.S3 Effect of graphene flakes on NIH-3T3 cell morphology. Representative confocal microscopy images of NIH-3T3 cells in the presence of graphene flakes (Scale bars, 50 μm). Graphene flakes (0.5-20 μg/mL) have insignificant impact on NIH-3T3 cell morphology. Cells were incubated for 24 h on poly-L-lysine coated coverslips and treated with graphene flakes, followed by staining with Acti-stain 488 phalloidin (actin, green) and DAPI (nucleus, blue).
